# Supplementary material for: Longitudinal Modulation of Loco-Regional Immunity in Ovarian Cancer Patients Receiving Intraperitoneal Chemotherapy
Source: Cancers (Basel). 2022 Nov 17;14(22):5647. doi: 10.3390/cancers14225647 (PMC9688312; doi:10.3390/cancers14225647)

Supplemental Table S1 IP fluid DE genes between early and late time points (p<0.05)

Gene	log2FC	p-value	q-value	Gene	log2FC	p-value	q-value	Gene	log2FC	p-value	q-value
SELE	4.165	1.26E-03	0.163	RRAD	2.219	0.014	0.180	IL7	2.162	0.029	0.196
CD70	3.637	1.56E-03	0.163	IL22RA2	2.567	0.014	0.180	TLR2	0.961	0.029	0.196
IL1R2	2.655	1.70E-03	0.163	FLT3LG	-1.929	0.015	0.180	CSF2	2.129	0.031	0.200
IL17B	4.231	1.85E-03	0.163	GZMB	-1.904	0.015	0.180	OSM	2.094	0.031	0.200
CD3EAP	3.167	1.86E-03	0.163	SPO11	3.524	0.015	0.180	PDCD1LG2	1.771	0.031	0.200
C6	3.966	2.03E-03	0.163	KIR3DL2	2.927	0.015	0.180	TFE3	1.796	0.031	0.200
TNFSF11	3.536	2.08E-03	0.163	CCL24	3.096	0.015	0.180	TNFSF4	1.751	0.032	0.203
CD209	2.912	2.41E-03	0.163	CCL17	2.550	0.016	0.180	CXCL13	1.983	0.033	0.205
TLR9	3.639	2.63E-03	0.163	IFNA2	2.804	0.016	0.180	RORC	2.106	0.033	0.205
TNFSF18	3.015	2.76E-03	0.163	MERTK	1.198	0.016	0.180	CD1A	2.194	0.033	0.206
MAPK11	3.880	2.81E-03	0.163	IL4	2.547	0.017	0.180	EGR1	1.749	0.034	0.206
IL5RA	3.671	2.91E-03	0.163	IRF2	1.571	0.017	0.180	IL2	2.483	0.034	0.206
CXCR1	3.309	3.24E-03	0.163	SMPD3	2.500	0.017	0.180	RAG1	2.139	0.034	0.206
IL22RA1	3.394	3.25E-03	0.163	CLEC6A	2.728	0.017	0.180	IL1RAPL2	2.149	0.035	0.207
CSF3	3.794	3.42E-03	0.163	ARG1	2.402	0.017	0.180	CASP10	1.670	0.035	0.209
C8G	3.223	3.66E-03	0.163	IL32	-1.333	0.017	0.180	C8B	2.079	0.036	0.209
IL17A	3.421	3.81E-03	0.163	AICDA	2.171	0.018	0.181	SELL	-1.299	0.036	0.209
IFNL1	3.243	4.01E-03	0.163	SEMG1	2.878	0.018	0.181	IL24	2.202	0.037	0.210
DMBT1	3.295	4.24E-03	0.163	MPPED1	2.728	0.018	0.184	TTK	1.999	0.037	0.210
MCAM	3.069	4.54E-03	0.166	LTK	2.518	0.019	0.184	RPS6	-1.360	0.037	0.210
THBS1	2.723	4.91E-03	0.168	NFATC4	2.367	0.019	0.184	C8A	2.037	0.038	0.211
IL1B	-2.961	5.11E-03	0.168	CREBBP	2.498	0.020	0.184	CD1B	1.932	0.038	0.211
TLR10	2.813	5.30E-03	0.168	IL22	2.961	0.020	0.184	FPR2	1.325	0.039	0.211
IL26	2.906	6.50E-03	0.178	CCL21	2.358	0.020	0.184	SERPINB2	1.662	0.039	0.211
ETS1	-1.608	6.85E-03	0.178	CCL1	2.237	0.021	0.184	TNFRSF17	2.082	0.039	0.211
CD3E	-1.910	7.17E-03	0.178	BAGE	2.558	0.021	0.184	CCL8	1.647	0.039	0.211
IL1RL2	3.021	7.74E-03	0.178	MASP1	2.451	0.021	0.184	CCR6	1.892	0.039	0.211
NCAM1	2.673	7.78E-03	0.178	PTGDR2	2.681	0.021	0.184	CD19	1.627	0.040	0.211
KIR3DL3	2.944	7.79E-03	0.178	MAGEA3	2.400	0.021	0.184	IFIT2	1.316	0.040	0.211

CCL16	3.191	7.96E-03	0.178	CD34	2.557	0.022	0.184	CXCR5	2.216	0.040	0.211
TPTE	3.224	8.33E-03	0.178	ROPN1	2.647	0.022	0.184	DEFB1	1.953	0.040	0.211
CR2	2.864	8.46E-03	0.178	C4BPA	2.936	0.022	0.184	FOXP3	1.765	0.040	0.211
IL8	-3.148	8.63E-03	0.178	TIRAP	2.104	0.022	0.184	IL3	2.495	0.042	0.214
IGLL1	3.073	8.85E-03	0.178	IL23R	2.313	0.022	0.184	JAM3	2.079	0.042	0.214
IL13	2.755	9.58E-03	0.178	BIRC5	1.951	0.023	0.188	CD274	2.155	0.042	0.214
BLK	2.833	9.60E-03	0.178	NFATC1	1.752	0.023	0.188	CCL11	1.977	0.042	0.214
MAGEA12	3.314	9.70E-03	0.178	NT5E	2.263	0.024	0.189	IL11	1.890	0.043	0.214
CRP	2.906	0.010	0.178	IL21	2.430	0.025	0.194	PBK	1.987	0.044	0.218
IL25	2.726	0.010	0.178	AMBP	2.372	0.025	0.194	IRGM	1.745	0.044	0.218
CCL22	2.368	0.010	0.178	IL27	2.085	0.025	0.194	EOMES	2.094	0.044	0.218
ZNF205	2.686	0.010	0.178	ELK1	1.890	0.025	0.195	TMEFF2	2.064	0.045	0.219
CCL18	2.046	0.011	0.178	SH2B2	1.891	0.026	0.196	MRC1	0.998	0.045	0.220
CMA1	2.677	0.011	0.178	CCL25	2.302	0.026	0.196	CDK1	1.723	0.046	0.220
IL15	2.300	0.011	0.178	IL5	2.511	0.027	0.196	ARG2	2.140	0.046	0.220
XCR1	2.253	0.011	0.178	IFIT1	1.460	0.027	0.196	C9	2.201	0.046	0.220
CD247	-1.721	0.011	0.178	FADD	2.362	0.028	0.196	PRF1	-1.288	0.047	0.220
AIRE	2.801	0.012	0.180	CCL28	2.339	0.029	0.196	MAGEC2	1.933	0.047	0.220
CTAG1B	2.648	0.012	0.180	NOS2A	2.220	0.029	0.196	CD79B	1.828	0.047	0.220
PASD1	2.504	0.012	0.180	IL1A	2.298	0.029	0.196	CCL15	2.226	0.047	0.220
GZMA	-1.579	0.013	0.180	TNFRSF13C	-2.016	0.029	0.196	CEACAM8	2.236	0.048	0.223
CCL19	2.497	0.013	0.180	MBL2	2.136	0.029	0.196	IL2RG	-0.967	0.049	0.223
MAGEA1	3.280	0.013	0.180	TNFRSF13B	2.367	0.029	0.196	SYCP1	2.216	0.050	0.226
CCR9	2.734	0.013	0.180	CXCR4	-1.482	0.029	0.196	PPBP	-2.229	0.050	0.226
CTCFL	2.675	0.013	0.180	LTF	2.362	0.029	0.196				

Supplemental Table S2 IP fluid DE genes between early and intermediate time points ($p<0.05$)

Gene	log2FC	p-value	q-value	Gene	log2FC	p-value	q-value
FCER1A	-3.379	5.94E-04	0.265	CXCR4	1.922	0.022	0.654
CXCL5	3.188	7.26E-04	0.265	CD160	-2.306	0.026	0.654
THBS1	2.555	1.18E-03	0.286	CCR2	-1.814	0.027	0.654
CD1C	-2.514	2.47E-03	0.438	POU2AF1	1.998	0.027	0.654
BLK	2.330	3.79E-03	0.438	IL4R	1.374	0.027	0.654
CXCL10	-3.704	4.38E-03	0.438	CD79B	1.743	0.027	0.654
CX3CR1	-2.535	4.76E-03	0.438	HLA-DPA1	-1.374	0.031	0.699
TNFRSF13C	2.747	4.80E-03	0.438	CXCL9	-2.451	0.032	0.699
CXCL12	-2.534	9.16E-03	0.547	TNFSF14	-1.957	0.034	0.699
CCL17	2.395	9.60E-03	0.547	CXCL11	-2.316	0.035	0.699
IL7R	2.333	9.83E-03	0.547	ITK	1.801	0.036	0.699
MS4A1	2.546	0.010	0.547	LY9	1.717	0.036	0.699
CD79A	2.625	0.011	0.547	TXK	1.510	0.039	0.699
C4B	-2.894	0.011	0.547	SERPINB2	1.664	0.041	0.699
CTLA4	2.037	0.011	0.547	MAGEB2	-1.771	0.041	0.699
FCER2	1.944	0.013	0.587	SIGLEC1	-1.543	0.041	0.699
C3	-2.587	0.015	0.617	CYBB	-1.305	0.042	0.699
ICOS	2.092	0.015	0.617	PPBP	-2.662	0.042	0.699
ISG20	1.692	0.017	0.640	LILRB3	-1.537	0.043	0.699
LILRA1	-1.908	0.018	0.640	HLA-DPB1	-1.229	0.043	0.699
CD19	1.653	0.019	0.640	TNFSF13	-1.330	0.045	0.699
CD22	1.636	0.020	0.640	TNFSF8	1.312	0.045	0.699
PAX5	1.944	0.020	0.640	AICDA	1.449	0.048	0.699
ETS1	1.778	0.022	0.654	TLR7	-1.375	0.049	0.699

Supplemental Table S3 IP fluid DE genes between intermediate and late time points ($p<0.05$)

Gene	log2FC	p-value	q-value	Gene	log2FC	p-value	q-value	Gene	log2FC	p-value	q-value
CXCR4	-3.166	2.05E-04	0.084	ZAP70	-1.995	0.018	0.284	IL1RL2	3.253	0.036	0.284
ETS1	-3.219	2.75E-04	0.084	IGLL1	3.795	0.019	0.284	CD1E	2.809	0.037	0.284
TNFRSF13C	-4.707	3.99E-04	0.084	CD1B	3.534	0.019	0.284	CMA1	3.094	0.037	0.284
CD3E	-3.222	4.58E-04	0.084	OAS3	1.909	0.019	0.284	CD70	3.079	0.038	0.284
IL7R	-3.184	6.49E-04	0.095	CXCR1	3.705	0.020	0.284	SEMG1	3.728	0.038	0.284
SELL	-2.902	1.29E-03	0.151	RORA	-2.322	0.020	0.284	IL5	3.202	0.038	0.284
CD79A	-3.538	1.58E-03	0.151	CD1A	3.360	0.020	0.284	TLR7	2.383	0.039	0.284
CD247	-2.682	1.77E-03	0.151	LTB	-1.973	0.020	0.284	IFNL1	2.976	0.039	0.284
FLT3LG	-3.301	1.90E-03	0.151	TNFSF18	3.055	0.020	0.284	DMBT1	3.130	0.039	0.284
TCF7	-3.059	2.07E-03	0.151	F13A1	1.802	0.020	0.284	CD80	2.520	0.039	0.284
CCR7	-3.309	2.61E-03	0.173	CD7	-2.481	0.021	0.284	CD3EAP	2.744	0.040	0.284
MS4A1	-3.296	2.91E-03	0.176	TLR4	1.534	0.022	0.284	TPTE	3.079	0.040	0.284
ITK	-2.853	3.14E-03	0.176	DEFB1	3.200	0.022	0.284	MAPK11	3.546	0.040	0.284
RPS6	-2.201	3.74E-03	0.195	MAGEA1	4.755	0.023	0.284	FPR2	1.998	0.040	0.284
CD1C	2.579	4.25E-03	0.200	TNFAIP3	-1.683	0.023	0.284	CYBB	1.515	0.041	0.284
ICOS	-3.455	4.39E-03	0.200	CSF2RB	2.012	0.024	0.284	C4BPA	3.901	0.041	0.284
CD3D	-2.500	5.23E-03	0.218	IL4	3.739	0.024	0.284	IL1R1	1.695	0.041	0.284
LY9	-2.779	5.44E-03	0.218	CYLD	-1.573	0.024	0.284	DUSP4	-1.923	0.041	0.284
CD48	-1.886	5.66E-03	0.218	TNFSF11	3.404	0.025	0.284	LTA	-2.153	0.041	0.284
SELE	5.366	6.87E-03	0.248	PASD1	3.188	0.025	0.284	TLR9	3.183	0.041	0.284
CD5	-2.698	7.19E-03	0.248	XCR1	2.832	0.025	0.284	RAG1	3.150	0.041	0.284
IL2RG	-1.712	7.47E-03	0.248	CD1D	2.163	0.026	0.284	IL4R	-1.309	0.042	0.284
IFIT1	2.549	9.76E-03	0.274	IL23R	3.330	0.026	0.284	IL17B	3.493	0.042	0.284
ITGA6	-2.456	9.77E-03	0.274	IL16	-1.455	0.026	0.284	IL9	3.208	0.042	0.284
CTLA4	-2.385	9.83E-03	0.274	NCAM1	2.923	0.027	0.284	SMAD3	-1.719	0.043	0.284
FYN	-1.602	0.010	0.274	CCL16	3.713	0.027	0.284	MAGEB2	2.559	0.043	0.284
JAK3	-2.339	0.011	0.274	CYFIP2	-1.700	0.027	0.284	IGF1R	-1.823	0.043	0.284
CD96	-1.989	0.011	0.274	CTCFL	3.751	0.028	0.284	IL17RB	3.541	0.044	0.284

FCER1A	3.014	0.011	0.274	LILRA1	2.546	0.029	0.284	MAGEA12	3.598	0.044	0.284
ANP32B	-1.533	0.013	0.284	CXCL6	2.065	0.029	0.284	ARG2	2.701	0.045	0.285
RUNX3	-1.957	0.014	0.284	HCK	1.465	0.031	0.284	LTF	3.108	0.046	0.285
IL22RA1	3.820	0.015	0.284	LCK	-1.824	0.031	0.284	IL15	2.524	0.046	0.285
THY1	2.029	0.015	0.284	IL5RA	3.511	0.032	0.284	STAT5B	-1.648	0.046	0.285
C3	2.211	0.015	0.284	IL17A	3.492	0.032	0.284	ROPN1	2.991	0.046	0.285
CD209	3.337	0.016	0.284	CD3G	-2.030	0.032	0.284	IL26	2.859	0.046	0.285
TNFSF14	3.186	0.016	0.284	CCND3	-1.587	0.033	0.284	PDCD1LG2	2.362	0.047	0.285
SIGLEC1	1.864	0.016	0.284	CXCL11	2.859	0.034	0.284	IFNG	2.664	0.048	0.285
CMKLR1	1.658	0.016	0.284	CASP8	-1.348	0.034	0.284	CSF2	2.891	0.048	0.285
CXCL10	3.080	0.016	0.284	MCAM	3.033	0.034	0.284	CLEC6A	3.275	0.048	0.285
CX3CR1	2.145	0.016	0.284	MEFV	3.012	0.034	0.284	CCL19	2.835	0.049	0.286
TXNIP	-1.532	0.017	0.284	IL6	2.821	0.036	0.284	KIR3DL3	2.853	0.049	0.286
SOCS1	-2.137	0.017	0.284	IL32	-1.384	0.036	0.284	C9	2.995	0.050	0.288

Supplemental Table S4 List of DE genes in IP fluid that are shared between time comparisons. Bolded genes have concurrent expression between time point comparisons

Three time comparisons	Early-Intermediate and Early-Late	Intermediate-Late and Early-Late		Early-Intermediate and Intermediate-Late
ETS1	**THBS1**	**SELE**	**CD247**	FCER1A
TNFRSF13C	**BLK**	**CD70**	**PASD1**	CD1C
CXCR4	**CCL17**	**IL17B**	**CCL19**	CXCL10
	**AICDA**	**CD3EAP**	**MAGEA1**	CX3CR1
	**SERPINB2**	**TNFSF11**	**CTCFL**	IL7R
	**CD19**	**CD209**	**FLT3LG**	MS4A1
	**CD79B**	**TLR9**	**IL4**	CD79A
	**PPBP**	**TNFSF18**	**CLEC6A**	CTLA4
		**MAPK11**	**IL32**	C3
		**IL5RA**	**SEMG1**	ICOS
		**CXCR1**	**ROPN1**	LILRA1
		**IL22RA1**	**C4BPA**	IL4R
		**IL17A**	**IL23R**	TNFSF14
		**IFNL1**	**IL5**	CXCL11
		**DMBT1**	**IFIT1**	ITK
		**MCAM**	**LTF**	LY9
		**IL26**	**CSF2**	MAGEB2
		**CD3E**	**PDCD1LG2**	SIGLEC1
		**IL1RL2**	**CD1A**	CYBB
		**NCAM1**	**RAG1**	TLR7
		**KIR3DL3**	**SELL**	
		**CCL16**	**RPS6**	
		**TPTE**	**CD1B**	
		**IGLL1**	**FPR2**	
		**MAGEA12**	**DEFB1**	
		**CMA1**	**ARG2**	
		**IL15**	**C9**	
		**XCR1**	**IL2RG**	

Supplemental Table S5 Combined IPA of DE genes in IP fluid identified the top canonical pathways in which these genes are involved for all three comparisons

Early-Late Comparison		Early-Intermediate Comparison		Intermediate-Late Comparison	
Pathway	p-value	Pathway	p-value	Pathway	p-value
Role of cytokines in mediating communication between immune cells	5.96E-29	Systemic lupus erythematosus in B cell signaling pathway	1.02E-11	Th1 and Th2 activation pathway	1.60E-22
Role of pattern recognition receptors in recognition of bacteria and viruses	1.63E-21	Primary immunodeficiency signaling	9.55E-10	Role of pattern recognition receptors in recognition of bacteria and viruses	1.71E-20
HMGB1 Signaling	1.95E-19	Granulocyte adhesion and diapedesis	3.94E-09	Crosstalk between dendritic cells and natural killer cells	3.79E-20
Altered T cell and B cell signaling in rheumatoid arthritis	3.17E-19	Agranulocyte adhesion and diapedesis	6.64E-09	Cardiac hypertrophy signaling (enhanced)	1.42E-19
Granulocyte adhesion and diapedesis	9.55E-19	Communication between innate and adaptive immune cells	5.18E-08	Systemic lupus erythematosus in B cell signaling pathway	1.93E-19
Cardiac hypertrophy signal	1.00E-18	PI3K Signaling in B Lymphocytes	4.47E-07	Th2 Pathway	1.58E-18
Hepatic Cholestasis	1.58E-18	Pathogenesis of Multiple Sclerosis	7.41E-07	Altered T Cell and B Cell Signaling in Rheumatoid Arthritis	2.00E-18
Systemic lupus erythematosus in B cell signaling pathway	5.01E-18	B Cell Development	9.77E-07	HMGB1 Signaling	3.98E-17
Role of macrophages, fibroblasts and endothelial cells in rheumatoid arthritis	6.31E-17	Altered T Cell and B Cell Signaling in Rheumatoid Arthritis	1.32E-06	Primary Immunodeficiency Signaling	5.01E-17
Agranulocyte adhesion and diapedesis	7.944E-17	B Cell Receptor Signaling	2.51E-06	Communication between Innate and Adaptive Immune Cells	2.00E-16

Supplemental Table S6 DE genes between pre-treatment and post-treatment tumor tissue (p<0.05)

Gene	log2FC	p-value	q-value	Gene	lof2FC	p-value	q-value
CXCL9	-2.451	0.002	0.497	IL19	1.481	0.026	0.497
CT45A1	-3.161	0.004	0.497	MASP1	1.734	0.026	0.497
ISG15	-2.541	0.005	0.497	IFNL1	1.435	0.026	0.497
STAT1	-1.915	0.005	0.497	IL4	1.896	0.026	0.497
SPP1	-2.987	0.005	0.497	IFNL2	1.797	0.029	0.497
CXCL10	-2.349	0.005	0.497	IL17A	1.815	0.031	0.497
IL5	2.048	0.007	0.497	KIR3DL3	1.701	0.032	0.497
CDK1	-1.632	0.007	0.497	IKBKE	-1.044	0.032	0.497
BST2	-1.901	0.007	0.497	LTA	1.460	0.034	0.497
PBK	-2.312	0.008	0.497	C4BPA	1.476	0.035	0.497
CCL21	-2.939	0.011	0.497	CMA1	1.450	0.036	0.497
MX1	-1.945	0.012	0.497	S100A7	1.523	0.036	0.497
CD24	-2.625	0.012	0.497	IL1RL1	1.440	0.036	0.497
CXCR4	-1.930	0.013	0.497	SPACA3	1.538	0.036	0.497
KIR_Inhibiting_Subgroup_2	1.886	0.013	0.497	AMBP	1.603	0.036	0.497
IL23A	1.972	0.014	0.497	KLRC1	1.361	0.037	0.497
CD22	-1.841	0.015	0.497	PPBP	1.550	0.038	0.497
PASD1	1.914	0.016	0.497	NFATC1	0.993	0.039	0.497
KIR_Inhibiting_Subgroup_1	1.799	0.017	0.497	KIR_Activating_Subgroup_2	1.511	0.040	0.497
IFIT1	-1.770	0.017	0.497	C8A	1.365	0.040	0.497
IL25	1.754	0.017	0.497	CXCR1	3.309	0.041	0.497
DDX58	-1.382	0.018	0.497	IL12B	1.484	0.041	0.497
CTAGE1	1.882	0.019	0.497	IFI27	-1.455	0.041	0.497
OAS3	-1.316	0.020	0.497	CD244	1.250	0.042	0.497
CCL1	1.797	0.020	0.497	FEZ1	0.907	0.042	0.497
GAGE1	1.736	0.020	0.497	DMBT1	1.442	0.043	0.497
CXCR2	1.799	0.022	0.497	LTB	-1.294	0.043	0.497
S100A8	1.876	0.023	0.497	MAGEC2	1.630	0.043	0.497

IL26	1.634	0.023	0.497	LCN2	-1.877	0.045	0.497
CCL7	1.705	0.024	0.497	CCL23	1.175	0.045	0.497
MAGEB2	1.698	0.024	0.497	CCL15	1.459	0.045	0.497
SH2D1B	1.623	0.025	0.497	LRRN3	1.144	0.045	0.497
MBL2	1.874	0.025	0.497	IFNA7	1.465	0.048	0.503
IDO1	-1.780	0.025	0.497	IRF7	-0.896	0.050	0.503

Supplemental Table S7 DE genes shared between early and late comparison in IP fluid and tumor tissue. Genes with concordant expression are bolded

Gene	
**CXCR1**	**C4BPA**
**IL17A**	**NFATC1**
**IFNL1**	**AMBP**
**DMBT1**	**IL5**
**IL26**	IFIT1
**KIR3DL3**	**MBL2**
**IL25**	**CXCR4**
**CMA1**	**C8A**
**PASD1**	PBK
**IL4**	CDK1
CCL21	**MAGEC2**
**CCL1**	**CCL15**
**MASP1**	PPBP

Supplemental Table S8 PBMC DE genes between early and late time points (p<0.05)

Gene	log2FC	p-value	q-value	Gene	log2FC	p-value	q-value
CXCL3	5.544	6.77E-04	0.150	APOE	2.330	9.42E-03	0.255
IL8	5.750	7.26E-04	0.150	THBS1	2.271	0.010	0.273
SPP1	5.931	7.50E-04	0.150	LAMP3	2.685	0.012	0.297
CXCL1	5.118	1.09E-03	0.150	IL24	2.198	0.013	0.311
CCL20	4.555	1.43E-03	0.150	USP9Y	0.880	0.016	0.357
IL1B	4.617	1.56E-03	0.150	NRP1	2.593	0.016	0.357
CXCL13	4.913	1.59E-03	0.150	CCL19	2.113	0.021	0.474
CCL22	4.408	1.91E-03	0.150	TNFSF10	-1.044	0.026	0.561
CCL2	5.672	1.93E-03	0.150	ENG	1.723	0.027	0.568
CXCL2	3.547	2.43E-03	0.150	IL1R1	1.562	0.029	0.568
CXCL5	3.875	2.47E-03	0.150	OSM	1.851	0.029	0.568
CCL24	4.245	2.63E-03	0.150	MME	1.626	0.030	0.568
IL1A	3.823	3.06E-03	0.150	IL19	1.841	0.030	0.568
IL1R2	2.789	3.09E-03	0.150	IFIT1	-1.464	0.032	0.586
SERPINB2	3.616	3.19E-03	0.150	CCL23	1.489	0.035	0.627
PLAU	3.530	3.47E-03	0.150	IL10	1.827	0.036	0.627
DUSP4	3.601	3.49E-03	0.150	C3	1.899	0.038	0.639
FN1	3.382	4.00E-03	0.158	CD83	1.387	0.039	0.639
IL6	2.836	4.11E-03	0.158	CXCR4	1.152	0.039	0.639
PPARG	1.774	5.15E-03	0.188	CXCL16	1.290	0.041	0.649
VEGFA	2.514	5.47E-03	0.190	NT5E	1.673	0.043	0.662
CTSL	3.202	5.82E-03	0.193	TNFAIP3	1.211	0.044	0.662
CLEC5A	3.457	6.81E-03	0.208	CCL3L1	1.537	0.044	0.662
PLAUR	1.931	7.18E-03	0.208	CCL3	1.572	0.046	0.666
CCL7	3.039	7.27E-03	0.208	IRAK2	1.460	0.047	0.680
IDO1	3.011	7.42E-03	0.208				

Supplemental Table S9 DE genes that are shared between early and late comparisons of IP fluid, tumor tissue and PBMCs. Genes with concordant expression are bolded

Three sample type comparison	IP fluid and PBMCs	Tumor tissue and PBMCs
CXCR4	**IL1R2**	SPP1
IFIT1	**THBS1**	CXCR4
	IL1B	**IFIT1**
	IL8	**CCL7**
	**CCL22**	IDO1
	**CCL19**	**IL19**
	**CCL24**	**CCL23**
	**NT5E**	
	IFIT1	
	**IL1A**	
	CXCR4	
	**OSM**	
	**CXCL13**	
	**IL24**	
	**SERPINB2**	

Supplemental Table S10 Combined IPA of DE genes for early and late time points of tumor tissue and PBMCs identified the top canonical pathways in which these genes are involved

Tumor tissue		PBMC	
Pathway	p-value	Pathway	p-value
Role of cytokines in mediating communication between immune cells	3.00E-14	Granulocyte adhesion and diapedesis	2.13E-31
Role of pattern recognition receptors in recognition of bacteria and viruses	5.31E-13	Agranulocyte adhesion and diapedesis	9.87E-31
Systemic lupus erythematosus in B cell signaling pathway	6.87E-13	TREM1 signaling	7.75E-14
Granulocyte adhesion and diapedesis	2.95E-12	Hepatic fibrosis/hepatic stellate cell activation	2.96E-13
Agranulocyte adhesion and diapedesis	6.30E-12	Role of IL17-A in arthritis	4.09E-13
Th1 and Th2 Activation Pathway	5.01E-11	Differential Regulation of Cytokine Production in Macrophages and T Helper Cells by IL-17A and IL-17F	1.58E-12
Altered T Cell and B Cell Signaling in Rheumatoid Arthritis	1.62E-10	Role of Macrophages, Fibroblasts and Endothelial Cells in Rheumatoid Arthritis	3.98E-12
Activation of IRF by Cytosolic Pattern Recognition Receptors	4.9E-10	Role of IL-17F in Allergic Inflammatory Airway Diseases	5.01E-12
Hepatic Cholestasis	2.45E-09	LXR/RXR Activation	6.31E-12
Cardiac Hypertrophy Signaling (Enhanced)	9.77E-09	Differential Regulation of Cytokine Production in Intestinal Epithelial Cells by IL-17A and IL-17F	1.00E-11

Supplemental Table S11 IP fluid DE genes in secondary cohort after one cycle of platinum based chemotherapy (p<0.05)

Gene	log2FC	p-value	q-value	Gene	log2FC	p-value	q-value	Gene	log2FC	p-value	q-value
TPSAB1	-4.450	1.78E-03	0.237	ENTPD1	2.190	0.018	0.237	TNFRSF17	2.095	0.031	0.237
CR2	3.473	2.56E-03	0.237	CSF2	2.511	0.018	0.237	IL5RA	2.022	0.031	0.237
SH2B2	2.746	2.59E-03	0.237	KIR3DL3	2.532	0.018	0.237	BLK	2.322	0.031	0.237
CCL13	2.985	3.09E-03	0.237	ICAM4	2.201	0.018	0.237	CD34	2.378	0.031	0.237
CCL18	2.513	3.20E-03	0.237	MR1	1.899	0.019	0.237	HAMP	1.807	0.032	0.237
IL34	3.439	3.36E-03	0.237	TMEFF2	2.467	0.019	0.237	PMCH	2.068	0.033	0.237
IL5	3.188	4.45E-03	0.237	IFNL1	2.735	0.019	0.237	CCL8	1.843	0.033	0.237
HSD11B1	3.416	4.53E-03	0.237	PLA2G6	2.026	0.020	0.237	ZNF205	2.186	0.034	0.237
CLEC6A	3.092	5.18E-03	0.237	IL11RA	2.263	0.020	0.237	IL13	2.366	0.034	0.237
TLR9	2.970	5.26E-03	0.237	PTGS2	1.938	0.020	0.237	CD40LG	1.649	0.034	0.237
MCAM	3.029	5.82E-03	0.237	PAX5	2.590	0.020	0.237	IL22RA2	2.206	0.034	0.237
MAPK11	3.109	6.78E-03	0.237	KIR_Inhibiting_Subgroup_1	2.146	0.020	0.237	LAIR2	1.500	0.034	0.237
ATM	2.914	6.94E-03	0.237	TNFRSF12A	2.155	0.021	0.237	NT5E	1.819	0.034	0.237
CREBBP	2.951	7.25E-03	0.237	C6	2.383	0.022	0.237	IFIT2	-1.558	0.035	0.237
CD70	2.616	7.58E-03	0.237	LBP	2.650	0.023	0.237	MAGEA12	2.243	0.035	0.237
CCL19	2.662	7.69E-03	0.237	CTCFL	2.435	0.023	0.237	CXCL9	1.705	0.036	0.237
CCL28	2.895	8.37E-03	0.237	IL12A	2.360	0.023	0.237	MAGEC2	2.302	0.036	0.237
MAGEC1	3.101	8.64E-03	0.237	TLR10	2.152	0.023	0.237	CLU	2.215	0.036	0.237
IL9	2.778	9.50E-03	0.237	MAGEA4	2.185	0.024	0.237	NCAM1	1.709	0.036	0.237
IL2	2.871	9.56E-03	0.237	ARG2	2.130	0.024	0.237	PASD1	2.338	0.037	0.237
CD79B	2.198	0.010	0.237	CDH5	2.107	0.024	0.237	IL3	2.346	0.038	0.237
IL15	2.302	0.010	0.237	IL24	2.375	0.025	0.237	SELE	2.409	0.038	0.237
PPBP	2.462	0.011	0.237	OSM	1.910	0.025	0.237	NFATC4	2.087	0.038	0.237
PDGFRB	2.591	0.011	0.237	BTLA	1.752	0.025	0.237	USP9Y	2.166	0.038	0.237
TNFRSF9	2.277	0.012	0.237	IL10	1.605	0.026	0.237	ECSIT	1.544	0.039	0.237
KIR_Inhibiting_Subgroup_2	2.271	0.012	0.237	CD1B	1.941	0.026	0.237	CMA1	2.325	0.039	0.237
MPPED1	2.769	0.012	0.237	NEFL	2.314	0.026	0.237	MME	1.896	0.039	0.237

NCR1	2.051	0.012	0.237	FLT3	1.899	0.026	0.237	TNFRSF11B	1.935	0.039	0.237
IL1RAPL2	2.562	0.014	0.237	IL1RL2	2.454	0.027	0.237	IFNL2	2.101	0.040	0.237
CSF3	2.497	0.014	0.237	IFNG	1.731	0.027	0.237	CCL1	2.250	0.040	0.237
FAS	2.074	0.014	0.237	EBI3	2.448	0.027	0.237	CLEC4C	1.642	0.040	0.237
TNFSF11	2.694	0.014	0.237	NOS2A	2.454	0.027	0.237	MAGEB2	1.726	0.041	0.238
IL12B	2.579	0.014	0.237	SPACA3	2.212	0.028	0.237	TAL1	2.254	0.041	0.238
JAM3	2.543	0.015	0.237	XCR1	1.899	0.028	0.237	SIGIRR	1.284	0.041	0.239
IL22	2.925	0.015	0.237	ITGA2B	2.294	0.028	0.237	CXCR2	-1.717	0.043	0.249
RUNX1	1.428	0.015	0.237	F12	2.260	0.028	0.237	IL11	2.102	0.044	0.250
C7	2.763	0.016	0.237	TNFRSF13B	2.412	0.028	0.237	BTK	1.315	0.045	0.250
AIRE	2.692	0.016	0.237	CCR6	1.993	0.029	0.237	IL17A	2.454	0.045	0.250
MBL2	2.841	0.016	0.237	IRGM	2.005	0.029	0.237	ATF1	1.152	0.045	0.252
ELANE	2.469	0.016	0.237	POU2AF1	2.250	0.029	0.237	CTAGE1	2.160	0.046	0.252
SYCP1	2.625	0.016	0.237	IL26	2.306	0.030	0.237	PPARG	1.697	0.046	0.252
ARG1	2.430	0.017	0.237	DDX43	2.378	0.030	0.237	S100A7	2.021	0.046	0.252
CHIT1	2.500	0.017	0.237	LILRA4	1.993	0.030	0.237	RRAD	1.939	0.047	0.254
ROPN1	2.473	0.017	0.237	FOXP3	1.840	0.030	0.237	CCL17	1.681	0.047	0.255
LTK	2.486	0.017	0.237	TNFRSF11A	1.668	0.030	0.237	MAP4K2	1.323	0.049	0.259
IL22RA1	2.589	0.017	0.237	TPTE	2.324	0.031	0.237				

Supplemental Table S12 Combined IPA of DE genes in early chemotherapy after one cycle of platinum-based chemotherapy identified the top canonical pathways in which these genes are involved

Pathway	p-value
Role of cytokines in mediating communication between immune cells	1.50E-23
Altered T cell and B cell signaling in rheumatoid arthritis	2.91 E-21
Role of pattern recognition receptors in recognition of bacteria and viruses	5.76E-20
Hepatic cholestasis	8.39E-20
HMGB1 signaling	5.50E-18
Systemic Lupus Erythematosus In B Cell Signaling Pathway	1.26E-17
Communication between Innate and Adaptive Immune Cells	1.58E-17
Differential Regulation of Cytokine Production in Intestinal Epithelial Cells by IL-17A and IL-17F	3.98E-17
Cardiac Hypertrophy Signaling (Enhanced)	1E-16
Differential Regulation of Cytokine Production in Macrophages and T Helper Cells by IL-17A and IL-17F	3.16E-16

Supplemental Figure S1. Sensitivity analyses of DE genes with and without the inclusion of potential outliers. (A) Heat map of log2FC of IP fluid DE genes between early and late time points with and without P2 ($p<0.05$). (B) Heat map of log2FC of PBMC DE genes between early and late time points with and without P5 ($p<0.05$).

A

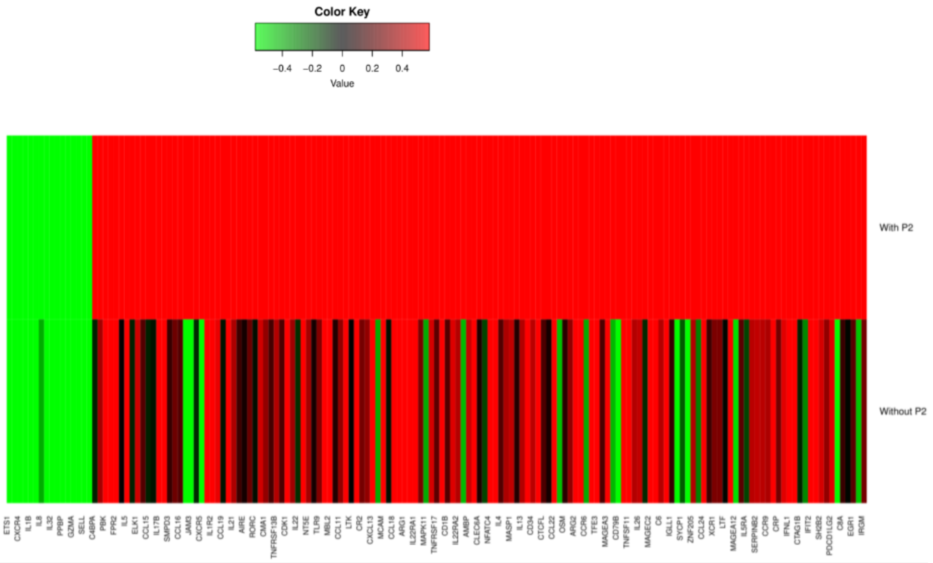

B

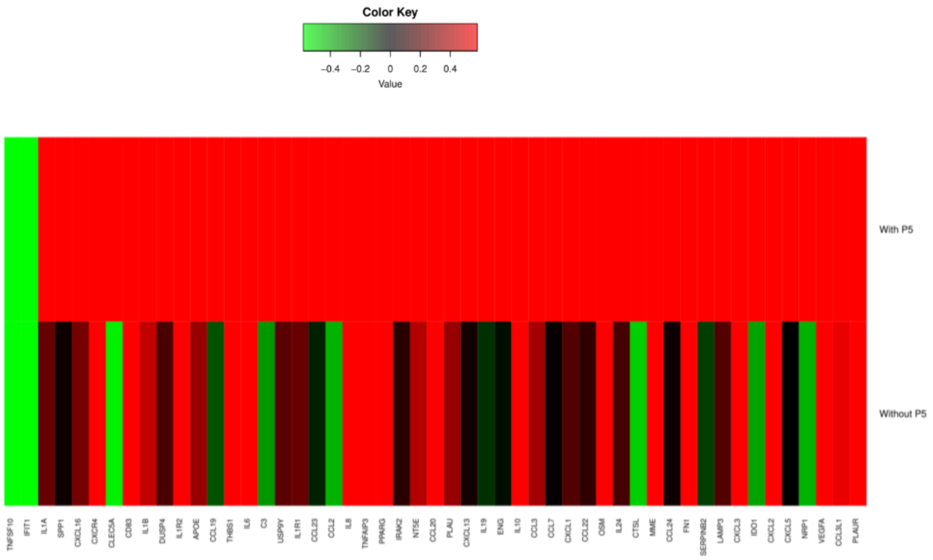

Supplement: Supplementary file 1 [file cancers-14-05647-s001.zip › cancers-1947990-SI.pdf]
